# Antiplasmodial and Leishmanicidal Activities of 2-Cyano-3-(4-phenylpiperazine-1-carboxamido) Quinoxaline 1,4-Dioxide Derivatives

**DOI:** 10.3390/molecules17089451

**Published:** 2012-08-07

**Authors:** Carlos Barea, Adriana Pabón, Silvia Galiano, Silvia Pérez-Silanes, German Gonzalez, Chloe Deyssard, Antonio Monge, Eric Deharo, Ignacio Aldana

**Affiliations:** 1 Research and Development of Novel Drugs Unit, Center for Applied Pharmacobiology Research (CIFA), University of Navarre, Pamplona 31080, Spain; Email: sgaliano@unav.es (S.G.); sperez@unav.es (S.P.-S.); amonge@unav.es (A.M.); ialdana@unav.es (I.A.); 2 Malaria Group, University of Antioquia, Medellín, Colombia; Email: apabon72@gmail.com; 3 UMR 152 PharmaDev (Pharmacologie et Pharmacochimie pour le Développement), Université de Toulouse, UPS, 118, rte de Narbonne, F-31062 Toulouse cedex 9, France; Email: germangonzalez.a@hotmail.com (G.G.); c.deyssard@gmail.com (C.D.); ericdeharo@gmail.com (E.D.); 4 UMR 152 PharmaDev Institut de Recherche pour le Développement, Faculté des Sciences Pharmaceutiques, Université de Toulouse 3, 31062 Toulouse, France

**Keywords:** quinoxaline, piperazine, *Plasmodium*, *Leishmania*, VERO

## Abstract

Malaria and leishmaniasis are two of the World’s most important tropical parasitic diseases. Thirteen new 2-cyano-3-(4-phenylpiperazine-1-carboxamido) quinoxaline 1,4-dioxide derivatives (CPCQs) were synthesized and evaluated for their *in vitro* antimalarial and antileishmanial activity against erythrocytic forms of *Plasmodium falciparum* and axenic forms of *Leishmania infantum*. Their toxicity against VERO cells (normal monkey kidney cells) was also assessed. None of the tested compounds was efficient against *Plasmodium*, but two of them showed good activity against *Leishmania*. Toxicity on VERO was correlated with leishmanicidal properties.

## 1. Introduction

Malaria is a major public health problem in more than 90 countries, affecting 40% of the World’s poorest population. Mortality due to malaria is estimated to be over 1 million deaths annually and this situation is worsened by the spread of drug-resistant strains of the parasite. Therefore, new effective and affordable antimalarial agents are urgently needed [1,2].

Leishmaniasis threatens approximately 350 million people and almost 12 million people are currently infected with the disease. The emergence of resistant parasites, the high cost and toxicity of current treatments call for the discovery of new drugs [3,4]. 

Quinoxalines, also known as 1,4-benzodiazines, are aromatic bicycles that present two nitrogen atoms on positions 1 and 4, described as bioisosteres of quinoline, naphtyl and some other heteroaromatic rings including pirazine [5].

Quinoxaline derivatives are of great interest as antimycobacterial, anti-inflammatory, anticancer and antiparasitic agents. More specifically, their 1,4-di-*N*-oxides are considered to be particularly important because they are responsible for a resulting increase in various biological properties [6].

As a result of different research projects, our group synthesized different series of quinoxaline 1,4-di-*N*-oxide derivatives, with a great variety of substituents in positions 2, 3, 6 and 7 [7,8,9,10,11,12,13,14,15]. With the aim of improving their pharmacological properties, we synthesized compounds with a carbonitrile group in position 2, thereby enhancing their antiparasitic activity. We also added an amine group in position 3 in order to link together new molecules, leading to interesting activities [10,16].

**Figure 1:**
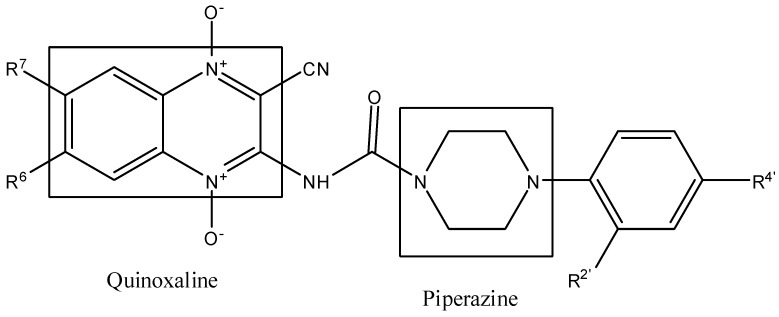
Design of new CPCQs as potential drugs against *P. falciparum* and *L. infantum*.

*In silico* studies showed that piperazine derivatives could target *Plasmodium* plasmepsin II enzyme. We synthesized phenylpiperazines derivatives that were active against *Plasmodium falciparum* [17]. In this context, we have now synthesized thirteen new 3-amino-1,4-di-*N*-oxide quinoxaline-2-carbonitrile derivatives linked with phenyl piperazines analogs (Figure 1) and investigated their *in vitro* activity and toxicity against *Plasmodium falciparum* Colombian FCR-3 strain and *Leishmania infantum* [18].

## 2. Results and Discussion

### 2.1. Chemistry

**Scheme 1:**
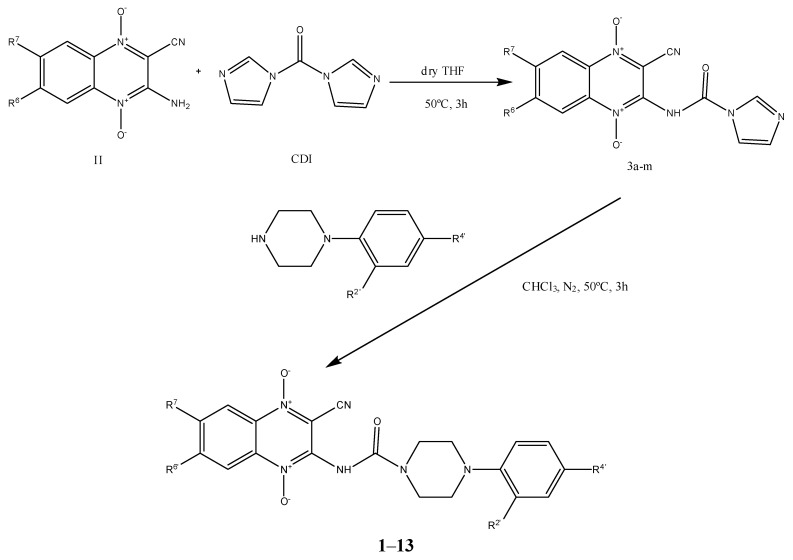
Synthetic route to CPCQs **1**–**13**.

We prepared thirteen new 2-cyano-3-(4-phenylpiperazine-1-carboxamido) quinoxaline 1,4-dioxide derivatives (CPCQs; Scheme 1). The benzofuroxane starting compounds, (BFX, **I**), were prepared using previously described methods [19,20]. The 3-amine-1,4-di-*N*-oxide quinoxaline-2-carbonitrile derivatives (cyanoamines, **II**) were obtained from the corresponding BFX by the Beirut reaction with malononitrile, using *N,N*-dimethylformamide (DMF) as solvent and triethylamine as catalyst [21].

The method for synthesizing the final compounds consisted of two steps; the first one was the reaction of cyanoamines with an excess of commercially available 1,1'-carbonyldiimidazole (CDI) in order to obtain the intermediates **3a**–**m** that can react in the second step with the phenylpiperazines, thus affording the CPCQs [22].

### 2.2. Pharmacology and Structure-Activity Relationship

With regard to antiplasmodial activity shown in Table 1, halogen groups in R^7^ and/or R^6^ increase the activity as shown in previous series of quinoxaline 1,4-di-*N*-oxide derivatives [16]. Compounds with CF_3_ or CH_3_O groups at R^4'^ show comparable activity, but higher activity than those which have F in that position. In relation with position R^2'^, the NO_2_ group does not increase the activity.

With regard to leishmanicidal activity shown in Table 1, among the most active compounds, **5** and **8** were also among the most cytotoxic compounds against VERO cells. Interestingly, the presence of halogen groups in R^7^ and/or R^6^, responsible for increasing anti-malarial activity, also increased leishmanicidal activity. Moreover, the compounds without NO_2_ group in R^2'^ showed considerably higher activity. Compounds with CF_3_ or F in R^4'^ show similar activity, but higher activity than those with CH_3_O in this position. 

**Table 1:** Biological characterization of the thirteen new quinoxaline 1,4-di-*N*-oxides.

Compd.	MW	R ^6^	R ^7^	R ^2'^	R ^4'^	IC_50_ (µM) a	IC_50_ (µM) b	CC_50_ (µM) c	SI ^d^
1	537.5	H	Cl	NO_2_	CF_3_	24.5	21.8	7.0	0.3
2	517	H	CH_3_	NO_2_	CF_3_	44.7	36.3	17.7	0.5
3	521	H	F	NO_2_	CF_3_	14.6	41.1	11.0	0.3
4	572	Cl	Cl	NO_2_	CF_3_	13.9	22.7	1.6	0.1
5	492.5	H	Cl	H	CF_3_	18.6	7.6	6.4	0.8
6	472	H	CH_3_	H	CF_3_	30.5	23.3	12.1	0.5
7	476	H	F	H	CF_3_	30.9	28.8	12.2	0.4
8	527	Cl	Cl	H	CF_3_	18.5	5.7	2.2	0.4
9	422	H	CH_3_	H	F	36.3	23.0	24.1	1.1
10	426	H	F	H	F	34.3	31.3	24.3	0.8
11	454.5	H	Cl	H	CH_3_O	12.8	18.8	47.5	2.5
12	434	H	CH_3_	H	CH_3_O	30.4	30.0	183.5	6.1
13	489	Cl	Cl	H	CH_3_O	26.1	10.9	14.0	1.3
CQ	320					0.1			
DOX	543.5						6.4	0.4	>10 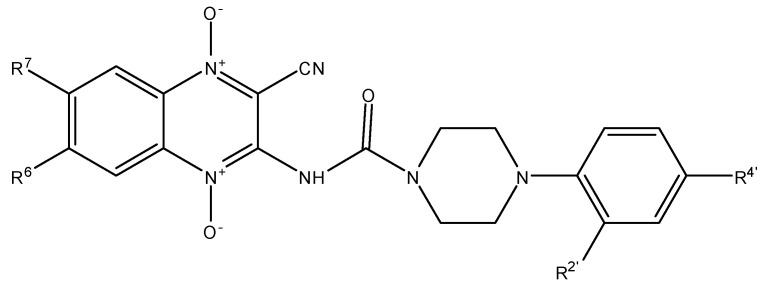

Selectivity index (SI): DT_50_ drug/IC_50_ drug. MW: molecular weight; ^a^ IC_50_ against *P. falciparum* FCR-3; ^b^ IC_50_ against *L. Infantum*; ^c^ Cytotoxicity in VERO cells; ^d^ Selectivity index.

## 3. Experimental

### 3.1. Chemical Synthesis

#### 3.1.1. General Remarks

All of the synthesized compounds were chemically characterized by thin layer chromatography (TLC), infrared (IR), proton nuclear magnetic resonance (^1^H-NMR), melting point and elemental microanalyses (CHN). Alugram SIL G/UV254 (0.2 mm layer, Macherey-Nagel GmbH & Co. KG., Düren, Germany) was used for TLC and silica gel 60 (0.040–0.063 mm, Merck, Darmstadt, Germany) was used for Flash Column Chromatography. The ^1^H-NMR spectra were recorded on a Bruker 400 Ultrashield instrument (400 MHz, Bruker, Billerica, MA, USA), using TMS as internal standard and with DMSO-*d*_6_ as solvent; the chemical shifts are reported in ppm (δ) and coupling constants (*J*) values are given in Hertz (Hz). Signal multiplicities are represented by: s (singlet), bs (broad singlet), d (doublet), t (triplet), q (quadruplet), dd (double doublet) and m (multiplet). The IR spectra were recorded on a Nicolet Nexus FTIR (Thermo, Madison, WI, USA) in KBr pellets. Elemental microanalyses were obtained on a CHN-900 Elemental Analyzer (Leco, Tres Cantos, Spain) from vacuum-dried samples. The analytical results for C, H and N, were within ± 0.5 of the theoretical values. Chemicals were purchased from Panreac Química S.A. (Barcelona, Spain), Sigma-Aldrich Química, S.A. (Alcobendas, Spain), Acros Organics (Janssen Pharmaceutical, Geel, Belgium) and Lancaster (Bischheim-Strasbourg, France).

#### 3.1.2. General Procedure for the Synthesis of Cyanoamines **II**

Malononitrile (18.0 mmol) was added to a solution of the appropriate benzofuroxane (**I**, 15.0 mmol) in DMF (10 mL). The mixture was allowed to stand at 0 °C. Triethylamine (1.5 mL) was added dropwise, and the reaction mixture was stirred at room temperature in darkness for 1 day. The resulting precipitate was filtered off and washed by adding diethyl ether, affording the target compound. The obtained red solid was used in the next step without further purification [10]. The yield of this reaction depends on the substituents in position 5 and 6 in the benzofuroxane.

#### 3.1.3. General Procedure for the Synthesis of 2-Cyano-3-(4-phenylpiperazine-1-carboxamido) Quinoxaline 1,4-Dioxide (CPCQs)

The corresponding cyanoamine (5.0 mmol) was reacted with a slight excess (1.5 equiv.) of 1,1'-carbonyldiimidazole (CDI) in dry tetrahydrofuran (40 mL) during 3hours at 50 °C. The solvent was removed *in vacuo*. The solid was then purified by column chromatography with toluene/dioxane (6:4) as the solvent; this solvent was subsequently removed *in vacuo*. The intermediate (2.5 mmol) was stirred with an excess of phenylpiperazine (1.2 equiv.) in chloroform during 3 h at 50 °C and under nitrogen atmosphere. Next, the solvent was removed *in vacuo* and the solid was collected and purified by column chromatography using dichlorometane/methanol (9:1). Finally, the solvent was removed *in vacuo* and the solid precipitated with cold diethyl ether, and filtered off to obtain a solid [22].

*7-Chloro-2-cyano-3-(4-(2-nitro-4-(trifluoromethyl)phenyl)piperazine-1-carboxamido) quinoxaline 1*,*4-dioxide* (**1**). Yield 30%; ^1^H-NMR δ ppm: 8.30 (s, 1H, H_11'_); 8.24 (d, 2H, H_2'_, *J*_2'−3'_ = 2.44 Hz); 8.22 (dd, 1H, H_9'_, *J*_9'−11'_ = 2.73 Hz, *J*_9'−8'_ = 3.84 Hz); 8.19 (dd, 2H, H_6'_, *J*_6'−2'_ = 2.96 Hz, *J*_6'−5'_ = 3.70 Hz); 8.11 (s, NH); 7.96 (dd, 2H, H_3'_, *J*_3'−5'_ = 8.95 Hz, *J*_3'−2'_ = 9.02 Hz); 7.90 (d, 1H, H_8'_, *J*_8'−9'_ = 2.01 Hz); 7.88 (d, 1H, H_8_, *J*_8−6_ = 2.68 Hz); 7.62 (d, 1H, H_6_, *J*_6−5_ = 9.27 Hz); 7.54 (d, 1H, H_5_, *J*_5−6_ = 8.40 Hz); 7.49 (dd, 2H, H_5'_, *J*_5'−3'_ = 4.21 Hz, *J*_5'−6'_ = 8.77 Hz). IR ν cm^−1^: 3,105 (w, NH); 2,211 (w, C≡N); 1,533 (s, C=O); 1,326 (s, N^+^O^−^). Anal. Calc. for C_21_H_15_N_7_O_5_F_3_Cl: C: 46.88%; H: 2.79%; N: 18.23%. Found: C: 46.74%; H: 2.94%; N: 18.18%.

*2-Cyano-7-methyl-3-(4-(2-nitro-4-(trifluoromethyl)phenyl)piperazine-1-carboxamido) quinoxaline 1*,*4-dioxide* (**2**). Yield 50%; ^1^H-NMR δ ppm: 8.21 (d, 1H, H_5_, *J*_5−6_ = 3.85 Hz); 8.20 (d, 1H, H_8_, *J*_8−6_ = 1.66 Hz); 8.17 (s, 1H, NH); 7.91 (d, 1H, H_6_, *J*_6−5_ = 2.04 Hz); 7.88 (d, 1H, H_8'_, *J*_8'−9'_ = 2.43 Hz); 7.51 (s, 1H, H_11'_); 7.49 (s, 1H, H_9'_); 3.69 (d, 2H, H_2'_, *J*_2'−3'_ = 2.66 Hz); 3.25 (dd, 2H, H_6'_, *J*_6'−5'_ = 2.65 Hz, *J*_6'−2'_ = 3.11 Hz); 3.11 (d, 2H, H_3'_, *J*_3'−2'_ = 5.27 Hz); 2.56 (dd, 2H, H_5'_, *J*_5'−3'_ = 2.79 Hz, *J*_5'−6'_ = 3.40 Hz); 2.54 (s, 3H, CH_3_-C_7_). IR ν cm^−1^: 3,098 (w, NH); 2,231 (w, C≡N); 1,549 (s, C=O); 1,328 (s, N^+^O^−^). Anal. Calc. for C_22_H_18_N_7_O_5_F_3_: C: 51.06%; H: 3.48%; N: 18.95%. Found: C: 51.28%; H: 3.46%; N: 19.03%.

*2-Cyano-7-fluoro-3-(4-(2-nitro-4-(trifluoromethyl)phenyl)piperazine-1-carboxamido) quinoxaline 1,4-dioxide* (**3**). Yield 15%; ^1^H-NMR δ ppm: 8.38 (dd, 2H, H_3'_, *J*_3'−5'_ = 4.94 Hz, *J*_3'−2'_ = 9.74 Hz); 8.20 (dd,2H, H_2'_, *J*_2'−6'_ = 2.68 Hz, *J*_2'−3_ = 3.37 Hz); 8.13 (d, 1H, H_11'_, *J*_11'−9'_ = 1.79 Hz); 8.11 (dd, 1H, H_9'_, *J*_9'−11'_ = 1.42 Hz, *J*_9'−8'_ = 2.01 Hz); 7.90 (d, 2H, H_5'_, *J*_5'−6'_ = 2.64 Hz); 7.88 (dd, 2H, H_6'_, *J*_6'−2'_ = 2.81 Hz, *J*_6'−5'_ = 1.47 Hz); 7.69 (dd, 1H, H_6_, *J*_6−8_ = 5.87 Hz, *J*_6−5_ = 9.23 Hz); 7.51 (d, 1H, H_8_, *J*_8−6_ = 2.62 Hz); 7.49 (d, 1H, H_8'_, *J*_8'−9'_ = 2.67 Hz); 7.44 (d, 1H, H_5_, *J*_5−6_ = 2.13 Hz); 3.55 (s, NH). IR ν cm^−1^: 3,108 (w, NH); 2,362 (w, C≡N); 1,533 (s, C=O); 1,321 (s, N^+^O^−^). Anal. Calc. for C_21_H_15_N_7_O_5_F_4_: C: 48.36%; H: 2.87%; N: 18.80%. Found: C: 47.92%; H: 2.94%; N: 18.31%.

*6-7-Dichloro-2-cyano-3-(4-(2-nitro-4-(trifluoromethyl)phenyl)piperazine-1-carboxamido) quinoxaline 1,4-dioxide* (**4**). Yield 20%; ^1^H-NMR δ ppm: 8.37 (d, 1H, H_11'_, *J*_11'−9'_ = 1.47 Hz); 8.31 (s, NH); 8.25 (d, 1H, H_8_, *J*_8−5_ = 1.98 Hz); 8.18 (d, 1H, H_5_, *J*_5−8_ = 2.41 Hz); 7.95 (dd, 1H, H_9'_, *J*_9'−11'_ = 3.31 Hz, *J*_9'−8'_ = 9.84 Hz); 7.88 (d,2H, H_2'_, *J*_2'−3'_ = 6.93 Hz); 7.73 (d,2H, H_6'_, *J*_6'−5'_ = 1.89 Hz); 7.51 (m, 2H, H_5'_); 3.59 (d,2H, H_3'_, *J*_3'−2'_ = 4.38 Hz); 1.09 (t, 1H, H_8'_, *J*_8'−9'_ = 6.73 Hz, *J*_8'−11'_ = 6.73 Hz). IR ν cm^−1^: 3,104 (w, NH); 2,240 (w, C≡N); 1,532 (s, C=O); 1,330 (s, N^+^O^−^). Anal. Calc. for C_21_H_14_N_7_O_5_F_3_Cl_2_: C: 44.05%; H: 2.44%; N: 17.13%. Found: C: 43.87%; H: 2.50%; N: 17.36%.

*7-Chloro-2-cyano-3-(4-(4-(trifluoromethyl)phenyl)piperazine-1-carboxamido) quinoxaline 1,4-dioxide* (**5**). Yield 65%; ^1^H-NMR δ ppm: 8.13 (t,1H, H_9'_); 7.99 (t, 1H, H_8'_, *J*_8'−9'_ = 9.06 Hz, *J*_8'−12'_ = 9.06 Hz ); 7.78 (dd, 1H, H_12'_, *J*_12'−8'_ = 3.86 Hz, *J*_12'−11'_ = 9.00 Hz); 7.62 (dd, 2H, H_6'_, *J*_6'−2'_ = 1.21 Hz, *J*_6'−5'_ = 8.84 Hz); 7.58 (s, NH); 7.54 (d, 2H, H_3'_, *J*_3'−2'_ = 8.50 Hz); 7.50 (dd, 1H, H_11'_, *J*_11'−9'_ = 2.30 Hz, *J*_11'−12'_ = 9.19 Hz); 7.41 (dd, 1H, H_6_, *J*_6−8_ = 8.44 Hz, *J*_6−5_ = 9.06 Hz); 7.10 (dd, 2H, H_5__'_, *J*_5'−3'_ = 8.39 Hz, *J*_5'−6'_ = 9.06 Hz); 6.74 (d, 1H, H_5_, *J*_5−6_ = 7.39 Hz); 3.57 (d, 1H, H_8_, *J*_8−6_ = 1.47 Hz); 3.10 (d, 2H, H_2'_, *J*_2'−3'_ = 5.43 Hz). IR ν cm^−1^: 3,107 (w, NH); 2,234 (w, C≡N); 1,524 (s, C=O); 1,333 (s, N^+^O^−^). Anal. Calc. for C_21_H_16_N_6_O_3_F_3_Cl: C: 51.16%; H: 3.24%; N: 17.05%. Found: C: 50.85%; H: 3.12%; N: 16.93%.

*2-Cyano-7-methyl-3-(4-(4-(trifluoromethyl)phenyl)piperazine-1-carboxamido) quinoxaline 1,4-dioxide* (**6**). Yield 23%; ^1^H-NMR δ ppm: 8.24 (d, 1H, H_5_, *J*_5−6_ = 8.08 Hz); 8.17 (d, 1H, H_6_, *J*_6−5_ = 6.82 Hz); 8.09 (s, 1H, H_11'_); 8.02 (s, 1H, H_9'_); 7.94 (s, NH); 7.82 (d, 1H, H_8'_, *J*_8'−9'_ = 9.78 Hz); 7.54 (d, 2H, H_2'_, *J*_2'−3'_ = 2.85 Hz); 7.53 (d, 2H, H_5'_, *J*_5'−6'_ = 4.83 Hz); 7.39 (d, 1H, H_12'_, *J*_12'−11'_ = 4.54 Hz); 7.32 (s, 1H, H_8_); 7.12 (d, 2H, H_3'_, *J*_3'−2'_ = 2.91 Hz); 7.10 (d, 2H, H_6'_, *J*_6'−5'_ = 1.88 Hz); 2.44 (s, 3H, CH_3_-C_7_). IR ν cm^−1^: 3,082 (w, NH); 2,233 (w, C≡N); 1,547 (s, C=O); 1,329 (s, N^+^O^−^). Anal. Calc. for C_22_H_19_N_6_O_3_F_3_: C: 55.93%; H: 4.02%; N: 17.79%. Found: C: 55.67%; H: 3.88%; N: 18.27%.

*2-Cyano-7-fluoro-3-(4-(4-(trifluoromethyl)phenyl)piperazine-1-carboxamido) quinoxaline 1,4-dioxide* (**7**). Yield 31%; ^1^H-NMR δ ppm: 7.89 (dd, 1H, H_5_, *J*_5-8_ = 2.75 Hz, *J*_5-6_ = 8.98 Hz); 7.71 (dd, 1H, H_6_, *J*_6−8_ = 2.75 Hz, *J*_6−5_ = 7.96 Hz); 7.67 (s, 1H, H_8_); 7.52 (dd, 2H, H_6'_, *J*_6'−2'_ = 7.83 Hz, *J*_6'−5'_ = 9.14 Hz); 7.45 (s, 2H, H_2'_); 7.38 (d, 1H, H_9'_, *J*_9'−8'_ = 8.55 Hz); 7.10 (dd, 2H, H_5'_, *J*_5'−3'_ = 8.94 Hz, *J*_5'−6'_ = 9.57 Hz); 6.97 (d, 1H, H_11'_, *J*_11'−12'_ = 9.11 Hz); 6.74 (dd, 2H, H_3'_, *J*_3'−5'_ = 7.94 Hz, *J*_3'−2'_ = 9.35 Hz); 3.70 (s, NH); 2.98 (d, 1H, H_12'_, *J*_12'−11'_ = 7.19 Hz); 2.59 (d, 1H, H_8'_, *J*_8'−9'_ = 6.56 Hz). IR ν cm^−1^: 3,106 (w, NH); 2,232 (w, C≡N); 1,532 (s, C=O); 1,332 (s, N^+^O^−^). Anal. Calc. for C_21_H_16_N_6_O_3_F_4_: C: 52.94%; H: 3.36%; N: 17.64%. Found: C: 52.53%; H: 3.32%; N: 17.76%.

*6,7-Dichloro-2-cyano-3-(4-(4-(trifluoromethyl)phenyl)piperazine-1-carboxamido) quinoxaline 1,4-dioxide* (**8**). Yield 17%; ^1^H-NMR δ ppm: 8.31 (s, 1H, H_5_); 7.88 (s, 1H, H_8_); 7.73 (d, 2H, H_6'_, *J*_6'−5'_ = 1.75 Hz); 7.54 (dd, 2H, H_3'_, *J*_3'−5'_ = 3.60 Hz, *J*_3'−2'_ = 9.32 Hz); 7.11 (dd, 2H, H_5'_, *J*_5'−3'_ = 4.68 Hz, *J*_5'−6'_ = 8.37 Hz); 6.74 (dd, 1H, H_8'_, *J*_8'−12'_ = 5.93 Hz, *J*_8'−9'_ = 8.95 Hz); 3.68 (dd, 1H, H_12'_, *J*_12'−8'_ = 2.93 Hz, *J*_12'−11'_ = 5.26 Hz); 3.57 (s, NH); 3.10 (dd, 2H, H_2'_, *J*_2'−6'_ = 3.92 Hz, *J*_2'−3'_ = 5.90 Hz); 2.59 (d, 1H, H_9'_, *J*_9'−8'_ = 1.89 Hz); 2.48 (dd, 1H, H_11'_, *J*_11'−9'_ = 1.55 Hz, *J*_11'−12'_ = 2.72 Hz). IR ν cm^−1^: 3,111 (w, NH); 2,364 (w, C≡N); 1,525 (s, C=O); 1,331 (s, N^+^O^−^). Anal. Calc. for C_21_H_15_N_6_O_3_F_3_Cl_2_: C: 47.81%; H: 2.84%; N: 15.93%. Found: C: 47.32%; H: 2.97%; N: 16.07%.

*2-Cyano-3-(4-(4-fluorophenyl)piperazine-1-carboxamido)-7-methylquinoxaline 1,4-dioxide* (**9**). Yield 79%; ^1^H-NMR δ ppm: 8.23 (d, 1H, H_5_, *J*_5−6_ = 8.39 Hz); 8.16 (s, 1H, H_9_); 7.94 (s, 1H, H_11_); 7.81 (d, 1H, H_6_, *J*_6−5_ = 8.46 Hz); 7.52 (d, 1H, H_8’_, *J*_8'−9'_ = 8.36 Hz); 7.39 (s, 1H, H_8_); 7.33 (s, 1H, NH); 7.17 (d, 1H, H_12'_, *J*_12'−11'_ = 8.17 Hz); 7.08 (d, 2H, H_2'_, *J*_2'−3'_ = 6.02 Hz); 7.06 (d, 2H, H_6'_, *J*_6'−5'_ = 7.13 Hz); 7.01 (d, 2H, H_3'_, *J*_3'−2'_ = 4.73 Hz); 6.99 (d, 2H, H_5'_, *J*_5'−6'_ = 6.17 Hz); 3.19 (s, 3H, CH_3_-C_7_). IR ν cm^−1^: 3,079 (w, NH); 2,231 (w, C≡N); 1,538 (s, C=O); 1,344 (s, N^+^O^−^). Anal. Calc. for C_21_H_19_N_6_O_3_F: C: 59.71%; H: 4.50%; N: 19.90%. Found: C: 59.80%; H: 4.55%; N: 19.51%.

*2-Cyano-7-fluoro-3-(4-(4-fluorophenyl)piperazine-1-carboxamido)quinoxaline 1,4-dioxide* (**10**). Yield 23%; ^1^H-NMR δ ppm: 8.41 (d, 1H, H_5_, *J*_5−6_ = 5.14 Hz); 8.38 (d, 1H, H_6_, *J*_6−5_ = 5.96 Hz); 8.14 (s, 1H, H_9'_); 8.12 (s, 1H, H_11'_); 7.91 (t, 2H, H_5'_, *J*_5'−6'_ = 8.92 Hz, *J*_5'−3'_ = 8.92 Hz); 7.45 (s, 1H, H_8_); 7.10 (s, 1H, H_8'_); 7.07 (s, 2H, H_2'_); 7.05 (s, 1H, H_12'_); 7.01 (d, 2H, H_3'_, *J*_3'−2'_ = 3.69 Hz); 7.00 (d, 2H, H_6'_, *J*_6'−5'_ = 3.25 Hz); 6.99 (s, 1H, NH). IR ν cm^−1^: 3,109 (w, NH); 2,231 (w, C≡N); 1,534 (s, C=O); 1,354 (s, N^+^O^−^). Anal. Calc. for C_20_H_16_N_6_O_3_F_2_: C: 56.33%; H: 3.75%; N: 19.71%. Found: C: 55.84%; H: 3.68%; N: 19.53%.

*7-Chloro-2-cyano-3-(4-(4-methoxyphenyl)piperazine-1-carboxamido)quinoxaline 1,4-dioxide* (**11**). Yield 12%; ^1^H-NMR δ ppm: 8.24 (s, 1H, H_8_); 8.19 (d, 1H, H_6_, *J*_6−5_ = 8.47 Hz); 8.13 (d, 1H, H_5_, *J*_5−6_ = 8.63 Hz); 8.07 (s, 1H, NH); 7.90 (d, 1H, H_9'_, *J*_9'−8'_ = 7.83 Hz); 7.63 (d, 1H, H_12'_, *J*_12'−11'_ = 6.55 Hz); 7.58 (s, 1H, H_11'_); 7.53 (d, 1H, H_8'_, *J*_8'−9'_ = 8.73 Hz); 6.93 (d, 2H, H_6'_, *J*_6'−5'_ = 6.87 Hz); 6.91 (d, 2H, H_2'_, *J*_2'−3'_ = 3.39 Hz); 6.84 (d, 2H, H_3'_, *J*_3'−2'_ = 7.41 Hz); 6.82 (d, 2H, H_5'_, *J*_5'−6'_ = 6.48 Hz); 3.63 (s,3H, CH_3_O). IR ν cm^−1^: 3,115 (w, NH); 2,228 (w, C≡N); 1,540 (s, C=O); 1,351 (s, N^+^O^−^). Anal. Calc. for C_21_H_19_N_6_O_4_Cl: C: 55.44%; H: 4.18%; N: 18.48%. Found: C: 54.97%; H: 3.95%; N: 18.21%.

*2-Cyano-3-(4-(4-methoxyphenyl)piperazine-1-carboxamido)-7-methylquinoxaline 1,4-dioxide* (**12**). Yield 32%; ^1^H-NMR δ ppm: 8.02 (d, 1H, H_5_, *J*_5−6_ = 8.69 Hz); 7.94 (d, 1H, H_8_, *J*_8−6_ = 2.45 Hz); 7.61 (dd, 1H, H_12'_, *J*_12'−8'_ = 1.78 Hz, *J*_12'−11'_ = 8.64 Hz); 7.52 (d, 1H, H_6_, *J*_6−5_ = 8.61 Hz); 7.39 (d, 2H, H_5'_, *J*_5'−6'_ = 3.60 Hz); 7.32 (s, 2H, H_6'_); 7.27 (dd, 1H, H_8'_, *J*_8'−12''_ = 1.61 Hz, *J*_8'−9'_ = 8.82 Hz); 6.92 (d, 1H, H_9'_, *J*_9'−8'_ = 8.44 Hz); 6.84 (d, 2H, H_3’_, *J*_3'−2'_ = 8.67 Hz); 3.69 (t, 3H, CH_3_O, *J*_7−8_ = 3.39 Hz, *J*_7−6_ = 3.39 Hz); 3.62 (s, 2H, H_2__'_); 3.13 (s, 1H, NH); 2.55 (s, 3H, CH_3_-C_7_); 1.23 (s, 1H, H_11__'_). IR ν cm^−1^: 3,120 (w, NH); 2,230 (w, C≡N); 1,546 (s, C=O); 1,328 (s, N^+^O^−^). Anal. Calc. for C_22_H_22_N_6_O_4_: C: 60.82%; H: 5.06%; N: 19.35%. Found: C: 61.20%; H: 4.61%; N: 19.84%.

*6-7-Dichloro-2-cyano-3-(4-(4-methoxyphenyl)piperazine-1-carboxamido)quinoxaline 1,4-dioxide* (**13**). Yield 11%; ^1^H-NMR δ ppm: 8.46 (d, 1H, H_8'_, *J*_8'−9'_ = 8.85 Hz); 8.29 (s, 1H, NH); 8.19 (d, 1H, H_12'_, *J*_12'−11'_ = 1.33 Hz); 7.87 (s, 1H, H_8_); 7.73 (d, 2H, H_6'_, *J*_6'−5'_ = 2.11 Hz); 6.94 (s, 1H, H_5_); 6.91 (s, 1H, H_9'_); 6.85 (s, 1H, H_11'_); 6.83 (s, 2H, H_2'_); 3.69 (s, 3H, CH_3_O); 3.18 (d, 2H, H_3'_, *J*_3'−2'_ = 3.00 Hz); 3.17 (d, 2H, H_5'_, *J*_5'−6'_ = 2.12 Hz). IR ν cm^−1^: 3,115 (w, NH); 2,229 (w, C≡N); 1,542 (s, C=O); 1,352 (s, N^+^O^−^). Anal. Calc. for C_21_H_18_N_6_O_4_Cl_2_: C: 51.53%; H: 3.68%; N: 17.17%. Found: C: 51.05%; H: 3.49%; N: 17.02%.

### 3.2. Pharmacology

#### 3.2.1. *In Vitro* Antiplasmodial Drug Assay

Chloroquine resistant FCR-3 strain of *P. falciparum* was cultivated at 37 °C in a 5% CO_2_ environment on glucose-enriched RPMI 1640 medium supplemented with gentamicin 0.1 mg/mL and 10% heat-inactivated A+ human serum, as previously described [23]. The drugs, dissolved in dimethylsulfoxide (DMSO), were added at final concentrations ranging from 250 to 0.1 µM. The final DMSO concentration was never greater than 0.1%. *In vitro* antimalarial activity was measured using the [3H]-hypoxanthine (MP Biomedicals, Santa Ana, CA, USA) incorporation assay [24]. Briefly, 250 µL of total culture medium with the diluted drug and the suspension of human red blood cell in medium (A^+^ group, 5% haematocrit) with 1% parasitaemia, were placed into the wells of 96-well microtitre plates. After 48 h of incubation at 37 °C in a 5% O_2_, 5% CO_2_ and 90% N_2_ atmosphere. On the third day of the test, radioactivity was assessed. All experiments were performed in triplicate. Results were expressed as the concentration resulting in 50% inhibition (IC_50_) which was calculated by a nonlinear regression logistic dose response model; the mean IC_50_ values and standard deviation for each compound was calculated.

#### 3.2.2. *In Vitro* Cytotoxicity

Toxicity was determined using Vero cells (normal monkey kidney cells) cultured under the same conditions as *P. falciparum*, except for the replacement of 5% human serum with 10% fetal calf serum. After the addition of compounds at increasing concentrations, cell growth was measured by [^3^H]-hypoxanthine incorporation after a 48-hour incubation period and then compared with a control sample [25].

#### 3.2.3.*In Vitro* Antileishmanial Drug Assay

Leishmanicidal activity was determined on axenic cultures of *L. infantum* amastigotes. In order to estimate the 50% inhibitory concentration (IC_50_) of the drugs, the 3-(4,5-dimethylthiazol-2-yl)-2,5-diphenyltetrazolium bromide (MTT) micromethod was used as previously described [26].

Briefly, Leishmania strain was maintained in promastigote stage in a biphasic medium (blood agar with 0.89% NaCl, pH 7.4) at 24 °C, with sub-passage every 3–4 days. Promastigotes (5 × 10^6^ parasites) were then transferred in M199 medium supplemented with 10% fetal bovine serum, pH 7.4. After 4 days, exponential phase promastigotes were centrifuged for 10 min at 1,500 g and 4 °C. The supernatant was discarded and replaced by fresh M199 medium supplemented with 20% FBS, pH 5.5. Axenic amastigotes transformation was then induced by increasing the temperature to 34 °C. Drugs were then tested at increasing concentrations.

## 4. Conclusions

All the tested compounds were almost 100 times less active against *Plasmodium* than chloroquine. Consequently they did not deserve further examination as antimalarials. Against *Leishmania* compounds **5** and **8** showed good activity and the most cytotoxic compound is four times less toxic than the reference drug. Unfortunately, these compounds show a low selectivity index. In this context, we suggest that synthesis should be focused on compounds with halogens at R^7^ and/or R^6^ in order to improve activity and lower toxicity.
